# South West Orthopaedic Club

**Published:** 1983-07

**Authors:** 


### South West Orthopaedic Club

Meeting at Southmead Hospital, Bristol, 27th November 1982

### ARE TWO KNIVES NOW REDUNDANT?
**John Fairclough, Cardiff**

By tradition in the orthopaedic world the surgeon uses two knives in his approach to the operation: (1) the skin knife; (2) the inside knife—on the presumption that the initial skin knife could be contaminated by skin bacteria, even after skin preparation has been performed. Mr. Fairclough had queried this premise particularly in the view of the high cost to the National Health Service and the inconvenience to the Theatre Sister and Surgeon in changing knives.

Mr. Fairclough had set up a research project in which they had examined the skin by taking pus swabs of the skin pre- and post-preparation, and taking bacterial culture of the skin knife and inside knife and bacterial examination of the wound. Of the 50+ patients examined and reviewed, 60% of the patients had contamination of the skin before the operation. However, no organism had been grown either from the pus swabs or from the blades. It was suggested, therefore, that there is no indication for the use of two scalpel blades in orthopaedic surgery.

H.R.

### THE FAILURE OF IODOPHOR SKIN PREP TO PREVENT CONTAMINATION OF CLEAN ORTHOPAEDIC WOUNDS BY SKIN ORGANISMS
**W. J. Mintowt-Czyz, Cardiff**

This study had shown that, in spite of effective disinfection of the skin surface by alcoholic betzidine, 15% of patients undergoing surgery for internal fixation of hip fractures grow skin organisms from the deep recesses of the wound prior to closure. It was suggested that the source of these organisms are the deep structures of the skin which are exposed when the skin incision is made.

H.R.

### THE PREVENTION OF LEG INJURIES IN MOTORCYCLE ACCIDENTS
**D. Ross, Perth**

Mr. Ross presented the results of his research, whilst working at the Bristol Royal Infirmary, into leg injuries in motorcycle accidents. He suggested that with improvements in both the construction and the acceptability of crash helmets, there will be more and more motorcyclists with severe lower limb injuries. He did, however, state that the number of motor cycles sold has not risen over the past 12 months. He illustrated the types of injuries which occur on the various forms of motorcycles and the possible forms of protection.

H.R.

### JEWETT NAIL PLATE OR A. O. DYNAMIC HIP SCREW FOR TROCHANTERIC FRACTURES? A RANDOMISED PROSPECTIVE CONTROLLED TRIAL
**G. C. Bannister and A. G. F. Gibson, Bristol**

The common complications of nail-plate fixation of trochanteric fractures are:

(1) Protrusion of the nail into the acetabulum.
(2) Cutting out of neck.
(3) Mechanical failure of the implant.

The previous two hospital comparisons have suggested that the sliding screw can reduce these complications from 25% to 6%. The reported reoperations rate for such complications is 29%.

Over a 13-month period from January 1981 to January 1982, 165 consecutive cases of trochanteric fractures of 65 years and over attending the Bristol Royal Infirmary and Southmead hospitals were randomly allocated to closed anatomical reduction and fixation with either the A.O. Dynamic hip screw or the Jewett nail plate. The present report covers results up to fracture union.

The dynamic hip screw permitted controlled collapse of the fracture by a mean of 15 mm compared with 3 mm in fractures fixed with the Jewett. Forty-four per cent of the Jewetts bent or broke, 10% patients requiring early reoperation. Ninety-five per cent of dynamic hip screws united satisfactorily with 5% cutting out. The dynamic hip screw has not failed to slide whereas the Jewett appears to distract the fracture from its natural tendency to collapse but be mechanically unequal to its task. Time of radiological union was similar.

There was no difference in mortality between those fixed with the Jewett and Dynamic hip screw.

Patients were awarded a functional score based on their independence, mobility and mode of walking preoperatively but there was no correlation between this and subsequent mortality.

Patients whose fractures were fixed within the first

24 hours had a slightly higher mortality (30%) than initially less fit patients who were given at least 1 day's medical treatment to improve their anaesthetic status. There was a lower mortality (15%) amongst patients operated by surgical staff performing the procedure frequently rather than occasional operators (40%), regardless of seniority.

Mortality with spinal anaesthesia (19%) was similar to that with inhalation methods (18%).

The A.O. Dynamic hip screw has been demonstrated to be a superior mechanical implant to the Jewett nail plate.

### THE SEMI-RIGID TOTAL HIP REPLACEMENT. IS THIS THE WAY FORWARD?

**K. J. J. Tayton and J. Bradley, Cardiff**

Most designs of total hip replacement have, in general, been very similar and although different protagonists defend their own particular variations, stainless steel femoral components and high density polyethylene acetabular components to be the norm.

Problems from stainless steel stems are now new, and consist of loosening, 'sinkage' fracture of the prosthesis, and fracture of the femoral shaft near the lower end of the prosthesis.

There are two approaches to solving these problems – the purely mechanical and the biological. Ling and Lee maintain that the solution is largely in prosthetic design and good cement fixation. However, although this may influence loosening, sinkage and prosthetic failure during the early years, it will not solve the problem of osteoporotic bone fracturing around a rigid implant.

Furthermore, bone splinted solid steel implant will undergo stress protection osteopenia, a process which occurs from the medulla outwards and which compounds the natural osteoporotic processes which also occur in the same area. Hence, however good a fixation is around a solid steel femoral stem, if the patients live long enough, then the femoral prosthesis is bound to come loose.

The solution is a semi-rigid femoral component which has the strength of a metal equivalent, but is much more flexible. With the flexibility offering much earlier transfer of load to the femoral shaft, osteopenia and hence sinkage and loosening cease to be such a problem (although of course natural osteoporosis still continues). If the material used has a very high resistance to fatigue stress, then implant failure should be eliminated, and as flexibility of the implant will be much closer to that of bone, fractures of the femoral shaft at the tip of the femoral stem should also be rare. Such a prosthesis has been designed by the authors. Details of the design, and the mechanical properties of this new hip, were discussed.

The satisfactory early results of clinical trials were presented.

### SOME OBSERVATIONS ON THE USE OF THE RICHARDS COMPRESSION SCREW PLATE IN THE TREATMENT OF INTERTROCHANTERIC HIP FRACTURES

**G. H. Heyse-Moore, A. G. MacEachern and D. C. Jameson-Evans, Exeter**

Two hundred patients with intertrochanteric fractures of the femur who could be followed either to union of the fracture or failure of treatment were studied. Half the patients had been treated with a Richards sliding screw/plate and half with a Jewett nail plate. The two groups were equivalent in terms of age, pre-operative medical condition, the grades of fracture and the experience of the operating surgeon.

Fixed angle nail plates have previously been shown to be unsatisfactory in the treatment of unstable fractures; we confirmed this finding by demonstrating a radiological failure rate of over two thirds in this group treated with a Jewett nail plate.

The stability of intertrochanteric fractures was discussed and it was shown that up to one third of supposedly stable two part fractures may in fact be unstable and our radiological failure rate of one third using the Jewett nail plate suggests that this is not a reliable method of treatment even in this apparently favourable group.

The use of the Richards compression screw plate is shown to have no greater early mortality or morbidity than the Jewett nail plate and the operating time is equivalent for the surgeon experienced in its use. The surgical technique including the use of the compression screw is discussed. This technique takes some time to acquire; however, the implant is shown to afford a degree of protection against technical error.

The results of treatment with the Richards screw plate are shown to be superior both clinically and radiologically to those using the Jewett nail plate, the number of poor clinical results being more than three times greater in this group. As a result of our experience we now feel that the reliability of the Richards implant makes it unnecessary to follow up the majority of patients after wound healing and discharge from hospital.

### A RECENT DEVELOPMENT IN FIXATION OF NECK OF FEMUR FRACTURES. THE SIEDEL VITALLIUM COMPRESSION HIP SCREW

**Anthony Mighell Smith, Taunton**

The compression hip screw system was described and the operative procedure illustrated. Emphasis was laid on early weight-bearing on the operated leg. Postoperative impaction and even displacement at the fracture site had been followed by sound bony union, so fracture comminution was not a contra-indication.

Forty-four patients treated with this device at Taunton from 8th October 1981 were compared with 44 matched patients treated the previous year with nail plates. There was no significant difference in the percentage of patients returning home or needing long-term care at 3 months after operation. There was little difference between the two groups as far as reduction in mobility was concerned at 3 months. The patients treated with compression hip screws, however, were generally able to leave hospital more quickly than those with nail plates.

Mechanical problems had been encountered in 5 patients. Four lag-screws had penetrated the femoral head as a result of operative malpositioning or the presence of pre-existent osteo-arthritis. One patient's bone had been so osteoporotic that the screw had failed to get a grip in the head.

The device was felt to be valuable where the degree of comminution at the femoral neck fracture made postoperative impaction likely, and also to rescue a nail plate failure.

